# Characterization and Phylogenetic Analysis of the Mitochondrial Genome of *Shiraia bambusicola* Reveals Special Features in the Order of Pleosporales

**DOI:** 10.1371/journal.pone.0116466

**Published:** 2015-03-19

**Authors:** Xiao-Ye Shen, Tong Li, Shuang Chen, Li Fan, Jian Gao, Cheng-Lin Hou

**Affiliations:** 1 College of Life Science, Capital Normal University, Beijing, People’s Republic of China; 2 Key Laboratory of Bamboo and Rattan Science and Technology of the SFA, International Centre for Bamboo and Rattan, Beijing, People’s Republic of China; University Paris South, FRANCE

## Abstract

*Shiraia bambusicola* P. Henn. is a pathogenic fungus of bamboo, and its fruiting bodies are regarded as folk medicine. We determined and analyzed its complete mitochondrial DNA sequence (circular DNA molecule of 39,030 bp, G + C content of 25.19%). It contains the typical genes encoding proteins involved in electron transport and coupled oxidative phosphorylation (*nad1-6* and *nad4L*, *cob* and *cox1-3*), one ATP synthase subunit (*atp6*), 4 hypothetical proteins, and two genes for large and small rRNAs (*rnl* and *rns*). There is a set of 32 tRNA genes comprising all 20 amino acids, and these genes are evenly distributed on the two strands. Phylogenetic analyses based on concatenated mitochondrial proteins indicated that *S*. *bambusicola* clustered with members of the order Pleosporales, which is in agreement with previous results. The gene arrangements of Dothideomycetes species contained three regions of gene orders partitioned in their mitochondrial genomes, including block 1 (*nad6-atp6*), block 2 (*nad1-cox3*) and block 3 (genes around *rns*). *S*. *bambusicola* displayed unique special features that differed from the other Pleosporales species, especially in the coding regions around *rns* (*trnR-trnY*). Moreover, a comparison of gene orders in mitochondrial genomes from Pezizomycotina revealed that although all encoded regions are located on the same strand in most Pezizomycotina mtDNAs, genes from Dothideomycetes species had different orientations, as well as diverse positions and colocalization of genes (such as *cox3*, *cox1-cox2* and *nad2–nad3*); these distinctions were regarded as class-specific features. Interestingly, two incomplete copies of the *atp6* gene were found on different strands of the mitogenomic DNA, a finding that has not been observed in the other analyzed fungal species. In our study, mitochondrial genomes from Dothideomycetes species were comprehensively analyzed for the first time, including many species that have not appeared in previous reports.

## Introduction

*Shiraia bambusicola* P. Henn. is an important pathogen and causative agent of bambusicolous disease, with negative effects on plant growth. *S*. *bambusicola* is a highly specific pathogen, usually confining infection to *Brachystachyum densiflorum* and related species in China and *Bambusa* species in Japan [1,2]. It is noteworthy that the fruiting bodies of this fungus are widely used in the southern part of China for remedying inflammation, apoplexy and sciatica. The corresponding sporophores or mycelium are usually rich in bioactive compounds, such as hypocrellins, which are photosensitizers that possess antibiotic, antitumor, antiviral and anti-inflammatory properties [3–12].

The corresponding position of *S*. *bambusicola* has been reclassified several times over one hundred years of taxonomic research. Dating back to 1900, the genus *Shiraia* was first recorded as one member of Nectriaceae, Hypocreales, Pyrenomycetes [13]. Two years later, *Shiraia* was anchored in the Hypocreaceae family based on the base of the larger fleshy stroma [14]. This viewpoint was popular for several decades, until the ascus was observed to not be unitunicate but was instead bitunicate, and *Shiraia* was transferred to the Loculoascomycetes class, the Pleosporales order, and the Pleosporaceae family [15]. As illustrated in the ninth edition of the fungal dictionary, *Shiraia* was characterized as a Dothideales species with an undetermined family affiliation [16]. In recent studies, the taxonomic position of *Shiraia* has been analyzed phylogenetically by DNA sequence analysis in combination with morphological evidence. Sequencing of the 18S rDNA and ITS-5.8S rDNA regions indicated that the genus *Shiraia* should belong to Phaeospheriaceae, Pleosporales [17]. Liu et al. [18] erected a new family Shiraiceae in Pleosporales to accommodate *Shiraia* based on the partial 28S nrDNA nucleotide sequence. It is noticeable that whether regarded as a genus or a family, there is only one representative species present in this group, and no distinct differences were found among fungal isolates from different bamboo hosts [17].

As one of the most important organelles in the cell, the mitochondria play a vital role in generating energy [19]. The origin of the mitochondrial genome dates back to DNA transposable elements from α-proteobacteria in a eukaryotic host cell [20–24], although most of the mtDNA coding genes have been transferred into the nuclear chromosomes during evolution [25]. Because of its high copy number, apparent lack of recombination, and rapid evolution, mitochondrial genomes (mitogenomes) are widely accepted as effective markers for evolutionary studies in the fungal kingdom [26–31]. Fungal mitogenomics have improved tremendously in recent years with the application of new sequencing technology, and the availability of mitochondrial genomes has allowed for the resolution of numerous questions regarding evolutionary history. For example, in February 2013 the largest fungal mitochondrial genome at that time was reported from *Agaricus bisporus*, with 135,005 bp [32]. Several months later, another mitogenome with 235,849 bp arose from *Rhizoctonia solani* [33]. Trans-splicing in organelles was first demonstrated from the fungal species *Gigaspora margarita* by analysis of the complete mitochondrial genome sequence [34]. A similar phenomenon of group I introns in mitochondria from *Gigaspora rosea* revealed an unusual feature: the effect of a third helper RNA fragment in trans. Studies of *Pneumocystis jirovecii*, an important opportunistic pathogen associated with AIDS and other immunodeficiency conditions, displayed a special arrangement of genes among the mitogenomes from closely related species. Thus, detailed analysis of mtDNA sequences raises the possibility of identifying new therapeutic targets [35]. Separate analyses of gene arrangements from the orders Sordariales [36] and Helotiales [37] displayed significantly different conservation patterns; furthermore, fungal mitogenomes exhibit remarkable variation between and within the major fungal phyla in terms of gene order, as demonstrated by a comparison of 38 complete mtDNA sequences published in previous reports [38].

The class Dothideomycetes contains more than 1900 species and is regarded as the largest and most diverse class of Ascomycete fungi [39]. The species are taxonomically classified into 11 orders: Capnodiales, Dothideales, Myriangiales, Hysteriales, Jahnulales, Mytilinidiales, Pleosporales, Botryosphaeriales, Microthyriales, Patellariales and Trypetheliales. Pleosporales is the most diverse fungal order in Dothideomycetes, occupying one quarter of all dothideomycetous species [40]. Species in this order occur in various habitats and were identified as one hundred and five generic types in multigene phylogenetic analyses [41].

Despite the fact that more than 165 fungal mitogenomes have been uploaded into the public database of NCBI and the dominant sequences can be attributed to mitochondria from Ascomycete, there are just two sequences published officially from the Dothideomycetes class: one from Capnodiales (*Mycosphaerella graminicola*) [42] and the other from Pleosporales [31].

The number of fungal mitogenomes that have been partially or completely sequenced is increasing. These published sequences have the potential to speed up the development of classification, evolution, genetics and breeding engineering for their corresponding mycetes. In this study, we have completed the novel mitochondrial genome sequence of *S*. *bambusicola*, as well as four additional mitogenomic sequences that were analyzed but have not appeared in previous reports. All of the referenced Dothideomycete species are well-known plant pathogens from various hosts, such as *Bipolaris maydis* from southern corn [43], *Leptosphaeria maculans* from oilseed rape [44], and *Phaeosphaeria nodorunm* from wheat [45], and have obtained great attention for their impact on the agriculture and forestry industries. Additionally, the hypocrellins from *S*. *bambusicola* (Dothideomycetes, Dothideales) was found to have a special structure of perylenequinonoid compounds that was also identified in *Cercospora* spp. (Dothideomycetes, Capnodiales) as cercosporin, *Elsinoë* spp. (Dothideomycetidae, Myriangiales) as elsinochromes, and *Cladosporium phlei* (Dothideomycetes, Capnodiales) as phleichrome [46]. It is anticipated that the findings of comparative analysis of mitochondrial genomes will contribute to the understanding of fungal evolutionary biology and enrich the knowledge of fungal infection and toxins from Dothideomycete pathogens.

## Materials and Methods

### Mitochondrial DNA Purification

Strain zzz816 of *S*. *bambusicola* was isolated from moso bamboo (*Phyllostachys edulis*) seeds as endophytic fungi and was previously morphologically identified and molecularly characterized by our lab [47]. Fungal cultures were recorded and deposited in the China Forestry Culture Collection Center (CFCC).

The mycelium from subcultured colonies were scraped from the surface of the agar and frozen in liquid nitrogen for mtDNA extraction. The DNAse treatment of the whole mitochondrial pellets and then the extraction of mtDNA were dependent on the instructions from the DNeasy Plant Mini Kit (Qiagen, Hilden, Germany) and Lang’s protocol [48]; nuclear DNA interference was assessed by PCR for the target regions of ITS rDNA [49].

### Illumina Sequencing, Scaffold Assembly, and Mitochondrial Genome Annotation

Total mitochondrial DNA of *S*. *bambusicola* was sequenced using Illumina Hiseq 2000, and the resulting reads were assembled into contigs using the CLC Genomics Workbench (CLCbio). Eight scaffolds were identified by a sequence similarity search using published fungal mitogenomes, and the resulting sequences were combined into a single circular DNA using PCR to bridge the intergenic gaps. The complete mitochondrial genome was reassured using PCR-based DNA sequencing.

The genomic clones from four other Dothideomycetes species (*Pyrenophor​a tritici-​repentis* P​t-1C-BFP (NW 002475730), *Leptosphaeria maculans* (FP929115), *Bipolaris maydis* C5 (AIDY01000067 and AIDY01000043) and *Neofusicoccum parvum* UCRNP2 (AORE01000551)) were screened by high levels of sequence identity with fungal mitogenomes referenced in the NCBI database. Fragmented contigs were separately assembled to approximate complete mitogenomic sequences; these contigs contained almost all of the protein coding genes, with a minimum of the genes encoding *nad1–6*, *nad4L*, *cob*, *cox1–3*, *atp6*, *rnl* and *rns*. Another two mitogenomes from *P*. *nodorum* (NC 009746) and *M*. *graminicola* (NC 010222) were downloaded from the NCBI public database; both have been analyzed in previous reports [31,42]. In order to assure the consistency of the data, only the larger contigs (over 10 kb), which displayed high similarity with the official sequence of fungal mitogenomes, were selected out and re-analyzed using the methods described below. Some revisions were referenced in published sequence information databases.

Protein coding genes were identified with MFannot [50] and errors were revised with BLASTp and BLASTx (NCBI). tRNA genes were screened using tRNAscan-SE [51], ARWIN [52], AGAGORN [53] and RNAWEASEL [54]. The results were regarded as reliable when genes were predicted by at least two methods. Non-coding regions and rRNA genes were identified manually using BLAST search and alignment with other reference sequences.

### Repeat Structure and Sequence Analysis

We utilized the REPuter [55] and Tandem Repeat Finder programs [56] to screen for dispersed and tandem repeats. Direct (forward), inverted (palindrome) and reverse repeats were composed of dispersed repeats, and the corresponding hamming distance was equal to 3 with a size of more than 30 bp. The advanced parameter of the Tandem Repeat Finder program was set at 2 (match), 7 (mismatch), and 7 (indels). The settings of the minimum alignment score and the maximum period size were 50 and 500, respectively. After the two programs were finished, we manually modified the redundant results of dispersed repeats and deleted tandem repeats with less than 15 bp.

### Phylogenetic Analysis

Amino acid sequences of the protein-encoding genes *atp6*, *cob*, *cox1*, *cox2*, *cox3*, *nad1*, *nad2*, *nad3*, *nad4*, *nad4L*, *nad5* and *nad6* were used for phylogenetic analysis. These sequences were found in the mitogenomes of 32 Ascomycete species (S1 Table) and were concatenated using DAMBE software version 5.2.13 [57]. The alignment was performed using Clustal version X 1.83, and amino acids sharing low homology were eliminated by Gblocks. Three species belonging to Saccharomycetales (*Candida albicans*, *Ogataea angusta* and *Pichia pastoris*) were used as outgroup taxa in the phylogenetic analysis. For the Bayesian analysis, cpREV with the Akaike information criterion (AIC) was used to choose a substitution model for the concatenated dataset. The model GRT + I + G was chosen for the combined sequences. The Bayesian analysis was performed with MrBayes 3.1.2 [58,59] with two sets of four chains (one cold and three heated) and the STOPRULE option in effect, halting the analyses at an average standard deviation of split frequencies of 0.01. The sample frequency was set to 100, and the first 25% of trees were removed as burn-in. Bayesian posterior probabilities (PP) were obtained from the 50% majority rule consensus of the remaining trees. Clades receiving PP ≥ 99% were considered to be significantly supported.

### GenBank Accession Number

The *S*. *bambusicola* mitogenome sequence was deposited in GenBank under accession number (KM382246). The other mtDNA sequences were downloaded from reference sequences in the NCBI database (S1 Table).

## Results

### Mitochondrial Genome Description

The mitochondrial genome of *S*. *bambusicola* was sequenced using Illumina Hiseq 2000, and eight scaffolds were assembled into a typical circular DNA molecule with a length of 39,030 bp using PCR amplification to successfully span all gaps. The sequence was AT-rich, with an overall G+C content of only 25.19% (Table 1). Protein-coding gene regions had a G+C content of 27.1%, and RNA genes had a slightly higher GC content of 35.4%. In general, the mitochondrial genome of *S*. *bambusicola* was compact, with 68.96% of the genome containing coding regions.

**Table 1:** General features in the mitochondrial genome of *Shiraia bambusicola*.

Genomes features	Value
Genomes size (bp)	39,030
G+C content (%)	25.19
No. of protein-coding genes	17
G+C content of protein-coding genes (%)	27.1
Structural proteins coding exons (%)	47.33
No. of rRNAs/tRNAs	2/32
G+C content of RNA genes (%)	35.4
rRNAs+tRNAs (%)	18.92
Coding regions (%)	68.96
Intergenic regions (%)	30.51
No. of introns	1
No. of intronic ORFs	1
Introns (%)	3.24

**Fig 1:**
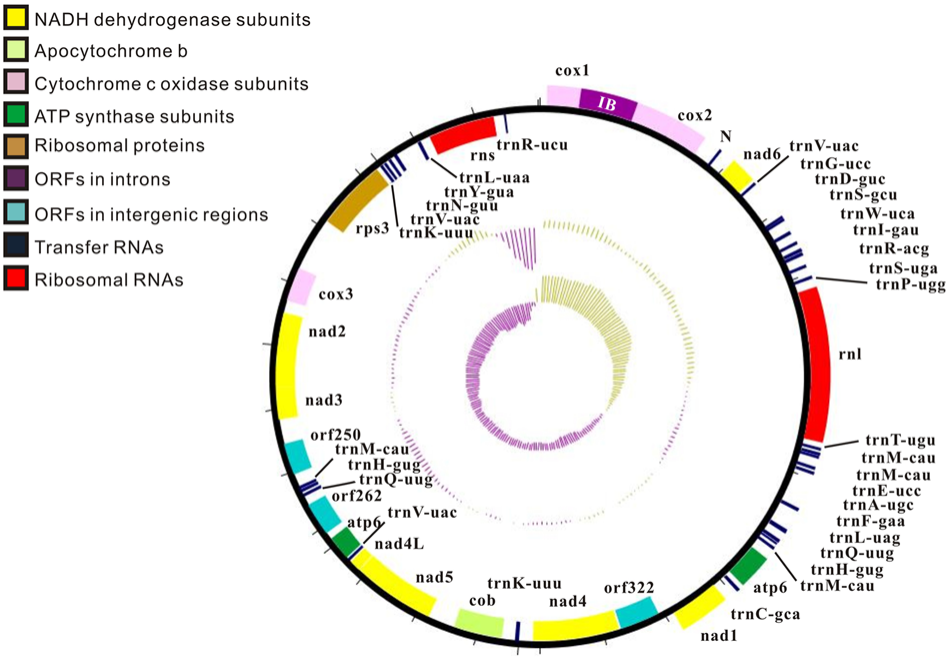
Circular mapping of the complete mitochondrial genome from *S*. *bambusicola*. The tracks from the outside represent: (1) Forward CDS and tRNA; (2) Reverse CDS and tRNA; (3) %GC plot (Yellow for above 50%, Violet for under 50%); (4) GC skew [(G − C)/(G + C)] (Yellow for plus, Violet for minus). The genes are colored on the basis of their functional groups, and the color scheme is illustrated to the left of the circle. One intron (IB) appeared in the corresponding gene *cox1*. The tRNA genes are indicated with brackets and the anticodon appended to the gene name. The precise positions of genes and introns are listed in Table 2.

Protein-coding gene regions accounted for 47.33% of the mitochondrial genome and contained 17 genes encoding proteins. These genes encoded for ATP-synthase complex F0 subunit (*atp6*), three complex IV subunits (*cox1*, *cox2*, and *cox3*), one complex III subunit (*cob*), seven electron transport complex I subunits (*nad1*, *nad2*, *nad3*, *nad4*, *nad4L*, *nad5*, and *nad6*), one ribosomal protein (*rps3*) and four hypothetical proteins (*orf250*, *orf262*, *orf322* and *orf352*) (Fig. 1) (Table 2). These genes appeared on both strands in an unbiased fashion. Thirteen representative mitochondrial genes involved in respiratory chain complexes (OXPHOS) displayed high sequence conservation with other species of filamentous fungi. Specifically, *cox1* was adjacent to *cox2* without intergenic regions, and the ATG initiation codon of *nad5* followed immediately after the termination codon of *nad4L*, with an overlap of one base.

**Table 2:** Gene organization of the mitochondrial genome.

Gene	Start position	Stop position	Length (nt)	Length (aa)	Start Codon	Stop codon
*cox1*	162	3046	2885	526	ATG	TGA
*cox2*	3047	3793	747	249	AAT	TAG
*trnN*	4169	4239	71			
*nad6*	4563	5129	567	189	ATG	TAA
*trnV*	5201	5273	73			
*trnG*	6116	6188	73			
*trnD*	6191	6262	72			
*trnS*	6502	6581	80			
*trnW*	6761	6832	72			
*trnI*	6934	7005	72			
*trnR*	7010	7081	72			
*trnS*	7288	7372	85			
*trnP*	7564	7636	73			
*rnl*	7808	11191	3384			
*trnT*	11299	11369	71			
*trnM*	11392	11462	71			
*trnM*	11468	11540	73			
*trnE*	11719	11791	73			
*trnA*	11824	11895	72			
*trnF*	12675	12747	73			
*trnL*	13201	13283	83			
*trnQ*	13459	13530	72			
*trnH*	13534	13607	74			
*trnM*	13655	13726	72			
*atp6*	13946	14719	774	258	ATG	TAA
*trnC*	14824	14895	72			
*nad1*	15187	16302	1116	372	ATG	TAA
*rps5*	17580	16612	969	323	ATG	TAA
*nad4*	19667	17622	2046	682	ATG	TAG
*trnK*	20096	20002	95			
*cob*	21573	20416	1158	386	ATG	TCC
*nad5*	24190	22205	1986	662	ATG	TAA
*nad4L*	24459	24190	270	90	ATG	TAA
*trnV*	24563	24491	73			
*atp6*	25228	24599	630	210	ATT	TAA
*orf262*	26126	25338	789	263	ATG	TAA
*trnM*	26415	26344	72			
*trnH*	26538	26465	74			
*trnQ*	26613	26542	72			
*orf250*	27677	26925	753	251	ATG	TAA
*nad3*	29026	28259	768	256	ATG	TAA
*nad2*	30778	29027	1752	584	ATG	TAA
*cox3*	31883	31074	810	270	ATG	TAA
*orf564*	34882	33188	1695	565	ATG	TAG
*trnK*	35050	34979	72			
*trnV*	35152	35080	73			
*trnN*	35288	35218	71			
*trnY*	35468	35384	85			
*trnL*	36120	36038	83			
*rns*	37944	36333	1612			
*trnR*	38242	38172	71			

Three open reading frames (ORFs) (*orf250*, *orf262*, *orf322*) were found in the intergenic regions and one ORF (*orf352*) was found in the intron. Most of the ORFs were located on the negative strand, with the exception of *orf352* in the intron of *cox1*. It is remarkable that there was only one group I intron across the entire mitochondrial genome, and only one intronic ORF encoding a putative LAGLIDADG endonuclease family protein with high similarity to other species of filamentous fungi, including *Talaromyces marneffei* and *Candida oxycetoniae*. The hypothetical protein encoded by *orf322* possessed some homology with YP 001427397, a ribosomal S5-like protein with a domain from the SNF7 superfamily, from *P*. *nodorum* (length = 323 aa; alignment range: 31–314 aa; identity = 67/291 (23%); e-value = 0.24). The *orf262* protein was slightly similar to an unnamed protein product with accession number XM 003024124 at the protein amino acid level; this protein is found in the nuclear genome of zoophilic dermatophytes *Trichophyton verrucosum* HKI 0517 (length = 263 aa; alignment range, 81–141 aa; identity = 27/75 (36%); e-value = 1.0). The *orf250* protein consisted of 251 amino acids, displayed no similarity with other fungal proteins, and was slightly similar to a hypothetical protein from *Thiohalocapsa* sp. by BLASTx (alignment range, 29–110 aa; identity = 45/83 (54%); e-value = 2.4).

The putative mitochondrial genes (*atp6*, *cob*, *cox1*, *cox2*, *cox3*, *nad1*, *nad2*, *nad3*, *nad4*, *nad4L*, *nad5*, *nad6*, *orf262*, *orf250* and *rpS3*) were applied to a study of the frequencies of codon usage for coding functional proteins. The codon usage of intronic genes was evaluated using the *orf352* sequence. The “AUG” initiation codon appeared most frequently (Table 3), with the exception of *cox2* with “AAU” and *atp6* with “AUU”. The intronic gene *orf352* started with special codon “UUG”, which was not found in other genes. The coding region of *cox1* was terminated by UGA and *cob* by UCC. *cox2* and *nad4* ended with UAG, and the stop codon used by all other genes was UAA, including intronic *orf352*.

**Table 3:** Codon usage of protein-coding genes in the mitogenome of *Shiraia bambusicola*.

AA		codon	%
Stop	End	UAA	7.03
K	Lys	AAA	5.29
Y	Tyr	UAU	4.92
F	Phe	UUU	4.9
I	Ile	AUU	4.84
I	Ile	AUA	4.43
N	Asn	AAU	4.37
L	Leu	UUA	2.89
R	Arg	AGA	2.79
F	Phe	UUC	2.74
S	Ser	AGU	2.55
I	Ile	AUC	2.47
Y	Tyr	UAC	2.42
T	Thr	ACU	2.37
C	Cys	UGU	2.29
W	Trp	UGA	2.29
S	Ser	UCU	2.18
T	Thr	ACC	2.14
H	His	CAU	2.03
N	Asn	AAC	2.01
S	Ser	AGC	1.95
E	Glu	GAA	1.77
L	Leu	UUG	1.69
V	Val	GUU	1.66
M	Met	AUG	1.62
S	Ser	UCC	1.38
V	Val	GUA	1.38
T	Thr	ACA	1.35
R	Arg	AGG	1.33
A	Ala	GCU	1.3
C	Cys	UGC	1.28
K	Lys	AAG	1.28
G	Gly	GGU	1.19
S	Ser	UCA	1.15
L	Leu	CUU	1.12
D	Asp	GAU	1.09
Stop	End	UAG	1.02
Q	Gln	CAA	0.97
P	Pro	CCU	0.78
G	Gly	GGA	0.71
A	Ala	GCA	0.7
H	His	CAC	0.55
L	Leu	CUC	0.55
W	Trp	UGG	0.55
T	Thr	ACG	0.45
P	Pro	CCC	0.42
P	Pro	CCA	0.41
V	Val	GUG	0.41
G	Gly	GGG	0.37
G	Gly	GGC	0.37
V	Val	GUC	0.36
L	Leu	CUA	0.29
L	Leu	CUG	0.23
R	Arg	CGU	0.23
D	Asp	GAC	0.19
Q	Gln	CAG	0.16
R	Arg	CGC	0.16
E	Glu	GAG	0.13
A	Ala	GCC	0.11
A	Ala	GCG	0.1
R	Arg	CGA	0.08
R	Arg	CGG	0.06
S	Ser	UCG	0.05
P	Pro	CCG	0.03

Table 3 shows that the most frequently used amino acid in the 17 protein genes was isoleucine, followed by serine (Table 3). As shown in Table 1, the mitochondrial genes are strongly biased toward codons with AT (72.9%), and the preference of A and U residues is consistent with overall codon usage. The most frequently used codons are composed exclusively of ‘‘U” and ‘‘A”: UAA (7.03%), AAA (5.29%), UAU (4.92%), UUU (4.90%), AUU (4.84%), AUA (4.43%), AAU (4.37%) and UUA (2.89%). The least frequent codons consist mainly of Cs and Gs (Table 3): CCG (0.03%), UCG (0.05%), CGG (0.06%), CGA (0.08%), GCG (0.10%) and GCC (0.11%).

tRNAscan-SE, ARWIN, AGAGORN, RNAWEASEL and BLAST comparison with other fungal mitochondrial genomes identified two rRNAs and 32 tRNAs in the genome, corresponding to all 20 amino acids (Table 4). Fig. 1 illustrates an ideogram that describes the genomic organization and gene classification; these genes are also located on both strands.

**Table 4:** tRNAs in the mitogenome of *Shiraia bambusicola*.

AA	Anticodon	Numbers
Ala	UGC	1
Arg	ACG	1
Arg	UCU	1
Asn	GUU	2
Asp	GUC	1
Cys	GCA	1
Gln	UUG	2
Glu	UUC	1
Gly	UCC	1
His	GUG	2
Ile	GAU	1
Leu	UAG	1
Leu	UAA	1
Lys	UUU	2
Met	CAU	4
Phe	GAA	1
Pro	UGG	1
Ser	GCU	1
Ser	UGA	1
Thr	UGU	1
Trp	UCA	1
Tyr	GUA	1
Val	UAC	3

In the *S*. *bambusicola* mitochondrial genome, 32 tRNAs were identified that clustered roughly into three groups (Fig. 1) with lengths ranging from 70 to 94 bp. The genes carried codons for all 20 amino acids, and some of them existed as multiple tRNAs (Table 4). There were four copies of the *trnM-CAU* tRNA gene for methionine and three tRNAs for valine with the same anticodon (*trnV-UAC*). Two different tRNA genes for leucine (*trnL-UAG* and *trnL-UAA*), arginine (*trnR-UCU* and *trnR-ACG*), and serine (*trnR-GCU* and *trnR-UGA*) were found. Two copies of *trnN-GUU*, *trnQ-UUG*, *trnH-GUG* and *trnK-UUU* were located in different regions; the remaining 11 tRNA genes had only one copy. It is noteworthy that two copies of three continuous genes of *trnQ-UUG*, *trnH-GUG* and *trnM-CAU* were separated by large distances on different DNA strands. All tRNAs exhibited the classic cloverleaf structure based on tRNAscan.

### Phylogenetic Relationships of Dothideomycetes

**Fig 2:**
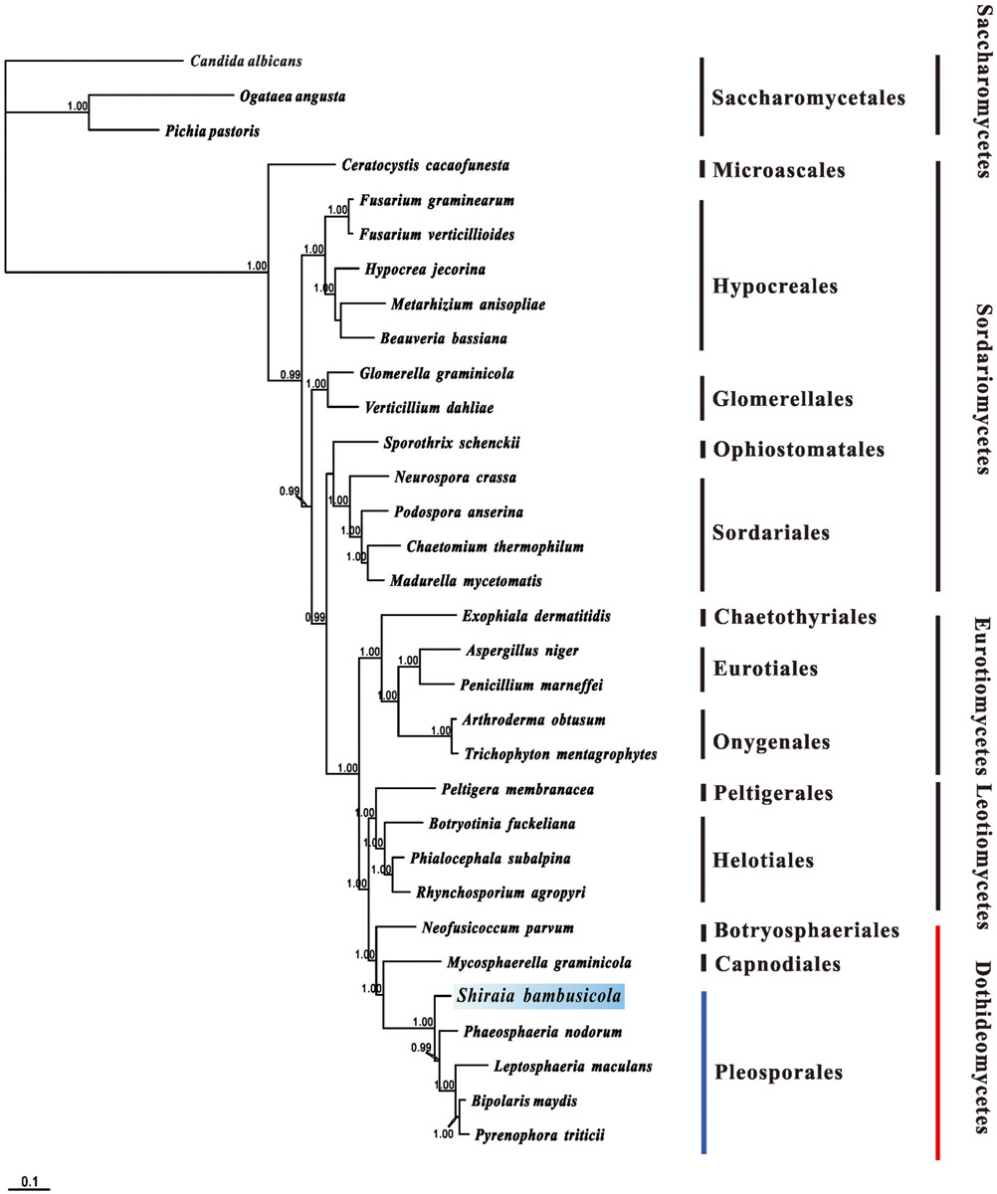
Phylogenetic tree from Bayesian analysis of amino acid sequences for Ascomycota mitochondrial genomes. The tree was based on 12 OXPHOS proteins: atp6, cob, cox1, cox2, cox3, nad1, nad2, nad3, nad4, nad4L, nad5 and nad6. Sequences were obtained from GenBank: *Bipolaris maydis* (AIDY01000067 and AIDY01000043), *Leptosphaeria maculans* (FP929115), *Pyrenophora tritici-repentis* (NW002475730), *Phaeosphaeria nodorunm* (NC009746), *Neofusicoccum parvum* (AORE01000551), *Mycosphaerella graminicola* (NC010222), *Beauveria bassiana* (NC010652), *Fusarium graminearum* (NC009493), *Fusarium fujikuroi* (NC016687), *Hypocrea jecorina* (NC003388), *Metarhizium anisopliae* (NC008068), *Cochliobolus heterostrophus* (JX185564), *Madurella mycetomatis* (JQ015302), *Chaetomium thermophilum* (NC015893), *Neourospora crassa* (KC683708), *Podospora anserine* (NC001329), *Sporothrix schenckii* (NC015923), *Glomerella graminicola* (CM001021), *Verticillium dahliae* (NC008248), *Annulohypoxylon stygium* (NC023117), *Penifillium marneffei* (NC005256), *Aspergillus niger* (NC007445), *Arthroderma obtusum* (NC012830), *Trichophyton mentagrophyte* (NC012826), *Exophiala dermatitidis* (CM001238), *Botryotinia fuckeliana* (KC832409), *Phialocephala subalpina* (NC015789), *Rhynchosporium agropyri* (NC023125), *Peltigera membranacea* (NC016957), *Candida albicans* (NC002653), *Pichia pastoris* (NC015384), and *Ogataea angusta* (NC014805). *Candida albicans*, *Pichia pastoris* and *Ogataea angusta* were used as the outgroups. Bayesian posterior probabilities were estimated and marked above the branches (≥ 99%).

In order to gain additional evidence for the classification of Dothideomycetes species and understand the evolutionary history of the mitochondrial genome, the complete concatenated amino acid sequences of the 12 standard mitochondrial genes (*atp6*, *cox1*, *cox2*, *cox3*, *nad1*, *nad2*, *nad3*, *nad4*, *nad4L*, *nad5*, *nad6* and *cob*) were used for phylogenetic construction by maximum parsimony (Fig. 2).

Using three species of Saccharomycetales in the class of Saccharomycetes (*Candida albicans*, *Ogataea angusta* and *Pichia pastoris*) as outgroups, four classes of Pezizomycotina species were identified (Dothideomycetes, Eurotiomycetes, Leotiomycetes and Sordariomycetes). In the Dothideomycetes group, the clade of five species belonging to Pleosporales were grouped separately from Botryosphaeriales and Capnodiales species, which clustered in the same clade associated with a posterior probability support of 95%. *S*. *bambusicola* was located amongst the species of the Pleosporales order with a high bootstrap support value of 100% and was a sister sequence to four other species in Pleosporales.

### Comparative View of Dothideomycetes mtDNAs

The sequenced mitochondrial genomes of Dothideomycetes showed remarkable variation in size, ranging from 39,030 bp (*S*. *bambusicola*) to over 154,863 bp (*L*. *maculans*) (S1 Table). The mitochondrial genome size of *S*. *bambusicola* (39,030 bp) was the smallest among the analyzed Dothideomycetes mtDNAs, including the orders Pleosporales, Botryosphaeriales and Capnodiales. The tremendous change in length can mainly be attributed to the variation in introns, intergenic regions and the presence of hypothetical proteins.

**Fig 3:**
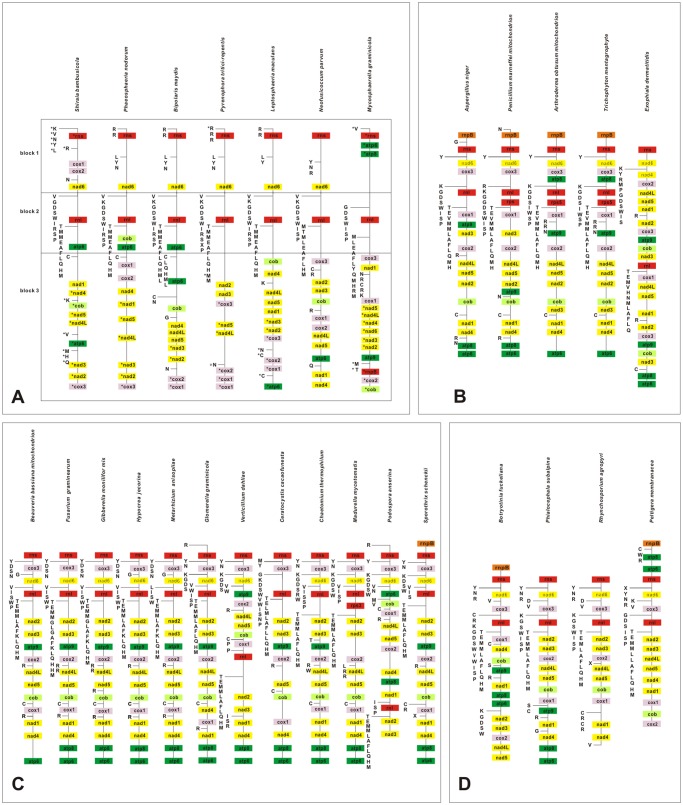
Mitochondrial gene orders of 29 fungal species from 4 classes. The classes are Dothideomycetes (A), Eurotiomycetes (B), Sordariomycetes (C) and Leotiomycetes (D). The genes are colored on the basis of their functional groups as in Fig. 1, and the positions of the tRNA genes are depicted using their one letter amino acid code. The noncoding region (NCR) is not indicated. An asterisk (*) indicates the opposite direction of transcription of genes.

As an effective tool to derive a common evolutionary route in fungi, mitochondrial genomes undergo complicated genome rearrangement. This gene order in *S*. *bambusicola* was compared with those of Dothideomycetes species whose mitogenomes have been sequenced and annotated completely or nearly completely (S1 Table). Seven mitogenomes were selected from the representative species: five in Pleosporlaes (*S*. *bambusicola*, *P*. *nodorum*, *B*. *maydis*, *P*. *tritici-repentis* and *L*. *maculans*), one in Botryosphaeriales (*N*. *parvum*) and one in Capnodiales (*M*. *graminicola*). As shown in Fig. 3, the sequences of protein-coding genes revealed significant areas of conservation and the gene order exhibited considerable synteny in the Dothideomycetes species, especially between Pleosporlaes species.

In the mitochondrial genome of *S*. *bambusicola*, the gene order could be identified for three representative regions, which include block 1 (*nad6*-*atp6*), block 2 (*nad1*-*cox3*) and block 3 (genes around *rns*) (Fig. 1). There was little diversity in gene arrangement in block 1 when compared with other Dothideomycetes species. In contrast, genes in block 2 underwent complex rearrangements among different species and the relative positions of the genes (*nad4*-*nad1*-*nad5*-*nad4L*) showed diverse patterns and different orientations. It is noteworthy that the block 3 organization of *S*. *bambusicola* was unique and differed from four adjacent species in the same order. Some specific genes were found to cluster together, indicating a strong relationship. For example, the gene pair *nad2*–*nad3* was permanently associated in all seven of the mitochondrial genomes analyzed, and no modifications were observed for gene pair *cox1*-*cox2* in six species (Fig. 3A).

To investigate if a similar pattern occurred in other closely related classes, the species of Eurotiomycetes, Leotiomycetes and Sordariomycetes were selected for genomic synteny analysis. Complete or nearly complete mitogenomes published from Pezizomycotina were limited to three classes, similar to our observations with Dothideomycetes. There was similar synteny in gene order between Chaetothyriales, Eurotiales and Onygenales (Fig. 3B). The parallel phenomenon remained when the species of Sordariomycetes were included in the analysis (Fig. 3C). Two subclasses (Hypocreomycetidae and Sordariomycetidae) did not show marked differences in gene arrangement. Two exceptions are the mitochondrial genome organization of *Verticillium dahliae* and *Podospora anserine*, which have special locations of *cox2*, *nad4L*, *nad5*, *nad4L*, *cob*, *nad4*, *cox1*, *nad1*, *atp8* and *atp6*. The mitogenome gene arrangement of the four species from Leotiomycetes also displayed a high degree of conservation in block 1 (*cox3*- *trnM*) and block 3 (genes around *rns*), while genes in block 2 exhibited variable order compared to different species (Fig. 3D). It is interesting that all genes for most of the known Pezizomycotina mtDNAs were encoded on the same strand with the exception of the Dothideomycetes species, and the diverse positions of *cox3* can be considered a class-specific feature. Among 29 mitogenomes from four classes, the gene arrangement of Eurotiomycetes and Sordariomycetes were the most conserved. For Leotiomycetes, four mitogenomes demonstrated three types of gene order in block 2. There were no regular arrangements of genes in block 2 of mtDNAs from Dothideomycetes, and some species also revealed unique locations of genes around *rns*. Furthermore, the distribution of mitogenomic genes in different strands improved the complexity of mitochondrial DNA sequences. In all, although more mitogenomes of Dothideomycetes species are required for an in-depth study, the existing open-source data allowed us to conclude that the mitochondrial gene orders in the class Dothideomycetes display more complex diversity than other species of Pezizomycotina.

### Introns and Intronic ORFs

Intronic elements in the mitochondrial genomes of Dothideomycetes species exhibit sequence variability, and intron insertion occurred irregularly in the coding genes (S2 Table). There were differences in the number of introns and in the length and content of intronic regions, particularly with regards to regions encoding open reading frames (ORFs).

Most fungal mitochondrial genomes that have been sequenced to date contain at least one group I and few group II introns. In the Pezizomycotina subphylum (including all published mitogenomes), the largest number of mitochondrial introns (n = 39) was documented for *P*. *tritici-​repentis* in our analysis (S2 Table), while *M*. *graminicola* is currently the only species of filamentous fungi entirely lacking mitochondrial introns (S2 Table) [42].

There was only one intron in the *cox1* gene sequence of *S*. *bambusicola*, and homologs of mobile elements were found to be inserted at a similar position (*cox1*) in *P*. *tritici-repentis*, *L*. *maculans*, *B*. *maydis* and *N*. *parvum* UCRNP2. These intronic ORFs (*orf352*, *orf324–1*, *orf324–2*, *orf318* and *orf321*) share high sequence identity (S3 Table). It is noteworthy that the existing introns of *cox1* genes from Dothideomycetes species were always found to contain these hypothetical proteins. The complete sequence of *orf324* appeared repeatedly in mitogenomes of *P*. *tritici-repentis* and *L*. *maculans*. There was no identical sequence from other species, including *B*. *maydis* in the same family (Pleosporaceae) as *P*. *tritici-repentis*. *L*. *maculans* intron II (domain V) from the *rns* gene also appeared at the same position in *N*. *parvum*, but their intronic sequences share low identity (S2 Table). It was interesting that the intronic ORFs encoding the genes appeared in the mitogenomes of *S*. *bambusicola* and *P*. *nodorum* less frequently and that similar mobile elements were usually found in other Dothideomycetes species. Many unidentified intronic ORFs encoding genes were found in *B*. *maydis*, *P*. *tritici-repentis*, *L*. *maculans* and *N*. *parvum*. Some of these were attributed to other fungal species, because the invasive ORFs exhibited higher comparative identity with the unknown proteins from distant relative species of filamentous fungi and even mushrooms.

### Unidentified Open Reading Frames and Conserved Open Reading Frames in the Intergenic Regions

The mitochondrial genome of *S*. *bambusicola* included functional genes that are generally found in other species; however, unique ORFs were identified in the intergenic regions of the unknown proteins. Three ORFs were detected in strain *S*. *bambusicola*, compared with 5 in *P*. *nodorum*, 64 in *B*. *maydis*, 36 in *P*. *tritici-repentis*, 28 in *L*. *maculans*, 8 in *N*. *parvum* and 15 in *M*. *graminicola*. These strains exhibited a broad spectrum of numbers of predicted ORFs, from the lowest ORF content (three in *S*. *bambusicola*) to the highest (64 in *B*. *maydis*). This variation in the number of predicted ORFs could partly explain the variation in genome size, and calculation of the percent identity of each genome revealed that most divergences were found in the intergenic regions.

In the mitogenome of *L*. *maculans*, one intergenic region contained an open reading frame (*orf221*). The putative homolog of this hypothetical protein was also found in the closely related *P*. *tritici-repentis* mitochondrial genome (*orf493*). Likewise, similar ORFs were discovered between the mitochondrial genomes of *L*. *maculans* (*orf207*, *orf535* and *orf158*) and *P*. *tritici-repentis* (*orf205* and *orf243*). It should be noted that although *orf221* (*orf493*) contained the conserved coding LAGLIDADG endonuclease region observed in other mycelial species (such as *Ceratocystis cacaofunesta* and *Annulohypoxylon stygium*), *orf158*, *orf205*, *orf207*, *orf243* and *orf535* sequences contained unique sequence structure features with no obviously matched regions detected in other species from the NCBI database.

### Other Notable Features

We found a number of repeats in the intergenic spacer (IGS) and coding sequence (CDS) regions of *S*. *bambusicola*, which were classified as 17 forward (direct), 22 inverted (palindromic), 6 reverse and 25 tandem repeats (S4 Table). Three repeats (P1, P2, and P3) were particularly long. P1 was the longest repeat with 485 bp and appeared in the CDS of *atp6*. Interestingly, there are only two partial copies of the *atp6* gene in the mitogenome, and neither of them encodes the complete *atp6* protein. P2 was located in the IGS region of the *trnL-trnQ* gene and the IGS of the *trnM- trnH* gene, and P3 appeared in the IGS sequences of *orf250*, *nad3* and *trnR*, and *cox1*. It is generally accepted that repeats can lead to genetic recombination, with the direct and inverted repeats represented during the loop-out process giving rise to submolecules, and a flip-flop mechanism giving rise to inversion.

In our study, the *atp8* and *atp9* genes only co-occurred in the mitogenome of *M*. *graminicola*, while another *atp9* gene was found in *N*. *parvum*. Neither the *atp8* or atp9 genes were identified from the mtDNA of five Pleosporales species; thus, only 12 genes coding for proteins related to oxidative phosphorylation without two ATP synthase subunits were used for phylogenetic studies on these species. It is noteworthy that a pair of *atp6* genes was found in the mitogenome of *S*. *bambusicola*; neither CDS could individually encode the complete ATP synthase F0 subunit 6, but the combination of the two fragments could assemble the complete gene. Their crossed region has a length of 543 bp, with the two partial genes appearing on different strands. This interesting phenomenon was verified by polymerase chain reaction (PCR) and conventional sequencing methods.

## Discussion

As one of the largest and most ecologically diverse classes of fungi, the comprehensive phylogenic reference data were derived from the combination of five genes (nucSSU, nucLSU rDNA, TEF1, RPB1 and RPB2) for 356 isolates in 41 families of Dothideomycetes [39]. Previously, 18 members of these species have been analyzed by comparing genome features. The order Pleosporales comprised more genes than Capnodiales, possibly implying the use of different modes of pathogenesis [60]. However, there are few reports of mitogenomic analysis. Although this approach is generally regarded as useful for evolutionary analysis, it has been applied only to Pleosporales and Dothideomycete species. To the best of our knowledge, this is the first study describing an intraspecific comparison of Dothideomycetes mitogenomes. We used seven complete or nearly complete mitogenomic sequences of Dothideomycetes species in this work: two (*P*. *nodorum* and *M*. *graminicola*) cited by published reports [31,42], four (*P*. *tritici-​repentis*, *L*. *maculans*, *C*. *heterostrophus* and *N*. *parvum*) assembled from contigs online, and one (*S*. *bambusicola*) sequenced by us. Analysis of mitogenomes from *P*. *tritici-​repentis*, *L*. *maculans*, *C*. *heterostrophus* and *N*. *parvum* was performed using sequence data downloaded from the NCBI database; thus, these sequences were not verified in this study (*S*. *bambusicola*) or previous studies (*P*. *nodorum* and *M*. *grami​nicola*). In future studies, PCR experiments should be applied to screening and correcting possible errors in the sequence data.

As illustrated in S1 Table, the mitochondrial genome of *S*. *bambusicola* displayed a circular DNA molecule with a length of 39,030 bp, which was the smallest of the closely related species. In contrast, the *L*. *maculans* mtDNA sequence comprised 154,863 bp. The various sizes were partly attributed to different intron and intergenic regions; for example, the intergenic region of the *S*. *bambusicola* mitogenome contains just one intron and three ORFs.

The phylogeny of *Shiraia* and related genera are still under debate, because the relative position of this group indicated special characteristics according to marker gene sequences (LSU rDNA, ITS regions and tub2) [17,61]. Recently, *Shiraia* species were deduced to be a new family anchored in the order of Pleosporales [18]. The phylogenetic tree based on mtDNA encoded proteins in our work suggested that four species grouped together as sister clades to *S*. *bambusicola* in the Pleosporales cluster. This new version of the relative position of *S*. *bambusicola* reinforces the hypothesis that *Shiraia* species should be included in the Pleosporales order.

Comparative analysis of gene arrangements is generally used to derive the evolutionary route. Here, we found that although *S*. *bambusicola* has been classified into the order of Pleosporales, the genomic organization of *S*. *bambusicola* differed considerably from other species (Fig. 3). Furthermore, the gene and tRNA order from Dothideomycete species seemed less conserved than other Pezizomycotina species (Eurotiomycetes, Leotiomycetes and Sordariomycetes). Protein coding genes and tRNAs appeared on different strands, which inferred a more complex organization of their relative positions. To investigate the high variability of mitochondrial gene order among Dothideomycetes species, further studies are required to exploit the additional mitogenomic sequences. tRNA genes that clustered as groups were generally regarded as a unique characteristic of fungal mitochondrial genomes [26], where the relevant contents and positions always display similar features in closely related species (Fig. 3). In the mitogenomes of Dothideomycetes species, the tRNA genes were distributed into three groups. There were large tRNA gene clusters around the *rnl* gene, a conserved pattern that also appeared in Eurotiomycetes, Leotiomycetes and Sordariomycetes species. The tRNA genes near *rns* contained a consensus RRLV from Pleosporales species. With the exception of special features contained by *S*. *bambusicola*, no similar order was found in other Ascomycete species. The analysis of mitogenomic sequences from Eurotiomycetes, Leotiomycetes and Sordariomycetes suggested that all genes were located on the positive strand, and arrangement of protein coding and tRNA genes displayed high sequence conservation, whether separately or reciprocally. It is remarkable that Dothideomycete species always contain several genes (such as *nad2*, *nad3* and *cox3*) located on different strands, especially in *M*. *grami​nicola* (Capnodiales) and *S*. *bambusicola* (Pleosporales), where the genes were distributed almost evenly between the two strands (especially *rns* and *rnl*). To the best of our knowledge, no similar pattern has been found in other Pezizomycotina mtDNA sequences. Kouvelis et al. [62] suggested that gene pairs *nad2-nad3*, *nad1-nad4*, *nad4L-nad5*, *atp6-atp8*, and *cob*-*cox1* usually remain joined in Ascomycetes, as was shown for Eurotiomycetes, Leotiomycetes and Sordariomycetes. However, in most mitogenomes present in Dothideomycetes species, the *atp8–9* genes were not present, and the *cytb*-*cox1* and *nad1*-*nad4* genes were uncoupled. Only two of these gene pairs were coupled (*nad2-nad3* and *nad4L-nad5*) on sections, and the *cox1-cox2* gene pair could be regarded as a typical trait for Dothideomycetes species, with the exception of *M*. *graminicola* from Capnodiales.

There are two genetic origins (nuclear and mitochondrial) for the fungal ATP synthase. The *atp6* gene from mitochondrial DNA usually encodes an essential subunit of the ATP synthase proton translocating domain, and we only identified two partial segments of duplications located in disperse positions of different strands from the mitogenome of *S*. *bambusicola*, For organelle genes, the case of trans-splicing has been reported in higher plants (such as wheat [63] and *Oenothera* [64]) and fungi (such as *Gigaspora rosea* [65]), but to the best of our knowledge, a similar case has not been described in ATP synthase genes from fungal species. Our further studies would focus on analysis of this gene expression and function to explore the internal mechanism.

As the largest order in the Dothideomycetes, Pleosporales included different species [40], most of which could be divided into epiphytes, endophytes or parasites of living leaves or stems, hyperparasites on fungi or insects, lichens, or saprobes of dead plant stems, leaves or bark [41,66]. Fungi belonging to the class Dothideomycetes are mostly soil-, wood- and dung-inhabiting fungi, and the seven described here belong to pathogens from living tissues of plants. *Shiraia* is a specific parasite of bamboo, infecting species of *Brachystachyum densiflorum*, *Bambusa*, and *Phyllostachys edulis*. We compared the ORFs of the intergenic regions and intronic ORFs of *S*. *bambusicola* with other species, including plant and animal fungi, and no potential mobile elements were found with high similarities. Seven Dothideomycete species used in this study are generally regarded as plant pathogens, and the complexity of gene arrangements in their mitogenomes inferred a possible impression from hosts to parasites. It is anticipated that further mitogenomic analyses would improve the understanding of plant-Dothideomycete pathogen interactions.

## Supporting Information

S1 Table Selected fungal species with published mitogenomes. (DOC)

S2 Table Introns found in mitochondrial genes from Dothideomycetes species. (DOC)

S3 Table ORFs found in mitochondrial genes from Dothideomycetes species. (DOC)

S4 Table Distribution of large repeat loci in the mitochondrial genome of *S*. *bambusicola*. (DOC)
