# Supplementary material for: Characterization and Phylogenetic Analysis of the Mitochondrial Genome of Shiraia bambusicola Reveals Special Features in the Order of Pleosporales
Source: PLoS One. 2015 Mar 19;10(3):e0116466. doi: 10.1371/journal.pone.0116466 (PMC4366305; doi:10.1371/journal.pone.0116466)
Supplement: S1 Table — (DOC) [file pone.0116466.s001.doc]

| **Table S1. Selected fungus species with published mitogenomes.** | | |  |  |  |
| --- | --- | --- | --- | --- | --- |
| **Species** | **Class** | **Order** | **Family** | **Size (bp)** | **Accession Number (Genbank)** |
| *Shiraia bambusicola* | Dothideomycetes | Pleosporales | Pleosporales incertae sedis | 39,030 | KM382246 |
| *Bipolaris maydis* | Dothideomycetes | Pleosporales | Pleosporaceae | 146,378 | AIDY01000067 and AIDY01000043 |
| *Leptosphaeria maculans* | Dothideomycetes | Pleosporales | Leptosphaeriaceae | 154,863 | FP929115 |
| *Pyrenophora tritici-repentis* | Dothideomycetes | Pleosporales | Pleosporaceae | 136,992 | NW002475730 |
| *Phaeosphaeria nodorunm* | Dothideomycetes | Pleosporales | Phaeosphaeriaceae | 49,761 | NC009746 |
| *Neofusicoccum parvum* | Dothideomycetes | Botryosphaeriales | Botryosphaeriaceae | 93,757 | AORE01000551 |
| *Mycosphaerella graminicola* | Dothideomycetes | Capnodiales | Mycosphaerellaceae | 43,964 | NC010222 |
| *Beauveria bassiana* | Sordariomycetes | Hypocreales | Cordycipitaceae | 29,961 | NC010652 |
| *Fusarium graminearum* | Sordariomycetes | Hypocreales | Nectriaceae | 95,676 | NC009493 |
| *Fusarium fujikuroi* | Sordariomycetes | Hypocreales | Nectriaceae | 53,753 | NC016687 |
| *Hypocrea jecorina* | Sordariomycetes | Hypocreales | Hypocreaceae | 42,130 | NC003388 |
| *Metarhizium anisopliae* | Sordariomycetes | Hypocreales | Clavicipitaceae | 24,673 | NC008068 |
| *Cochliobolus heterostrophus* | Sordariomycetes | Microascales | Ceratocystidaceae | 103,147 | JX185564 |
| *Madurella mycetomatis* | Sordariomycetes | Sordariales | mitosporic Sordariales | 45,590 | JQ015302 |
| *Chaetomium thermophilum* | Sordariomycetes | Sordariales | Chaetomiaceae | 127,206 | NC015893 |
| *Neourospora crassa* | Sordariomycetes | Sordariales | Sordariaceae | 64,840 | KC683708 |
| *Podospora anserina* | Sordariomycetes | Sordariales | Lasiosphaeriaceae | 100,314 | NC001329 |
| *Sporothrix schenckii* | Sordariomycetes | Ophiostomatales | Ophiostomataceae | 27,125 | NC015923 |
| *Glomerella graminicola* | Sordariomycetes | Glomerellales | Glomerellaceae | 39,649 | CM001021 |
| *Verticillium dahliae* | Sordariomycetes | Glomerellales | Plectosphaerellaceae | 27,184 | NC008248 |
| *Annulohypoxylon stygium* | Sordariomycetes | Xylariales | Xylariaceae | 133,782 | NC023117 |
| *Penifillium marneffei* | Eurotiomycetes | Eurotiales | Trichocomaceae | 35,438 | NC005256 |
| *Aspergillus niger* | Eurotiomycetes | Eurotiales | Aspergillaceae | 31,103 | NC007445 |
| *Arthroderma obtusum* | Eurotiomycetes | Onygenales | Arthrodermataceae | 24,105 | NC012830 |
| *Trichophyton mentagrophyte* | Eurotiomycetes | Onygenales | Arthrodermataceae | 24,297 | NC012826 |
| *Exophiala dermatitidis* | Eurotiomycetes | Chaetothyriales | Herpotrichiellaceae | 26,004 | CM001238 |
| *Botryotinia fuckeliana* | Leotiomycetes | Helotiales | Sclerotiniaceae | 82,212 | KC832409 |
| *Phialocephala subalpina* | Leotiomycetes | Helotiales | mitosporic Helotiales | 43,742 | NC015789 |
| *Rhynchosporium agropyri* | Leotiomycetes | Helotiales | mitosporic Helotiales | 68,904 | NC023125 |
| *Peltigera membranacea* | Leotiomycetes | Peltigerales | Peltigeraceae | 62,785 | NC016957 |
| *Candida albicans* | Saccharomycetes | Saccharomycetales | Saccharomycetaceae | 40,420 | NC002653 |
| *Pichia pastoris* | Saccharomycetes | Saccharomycetales | Saccharomycetaceae | 35,683 | NC015384 |
| *Ogataea angusta* | Saccharomycetes | Saccharomycetales | Saccharomycetales incertae sedis | 41,719 | NC014805 |
