# Supplementary material for: Characterization and Phylogenetic Analysis of the Mitochondrial Genome of Shiraia bambusicola Reveals Special Features in the Order of Pleosporales
Source: PLoS One. 2015 Mar 19;10(3):e0116466. doi: 10.1371/journal.pone.0116466 (PMC4366305; doi:10.1371/journal.pone.0116466)
Supplement: S2 Table — (DOC) [file pone.0116466.s002.doc]

| **Table S2. Introns found in mitochondrial genes from Dothideomycetes species.** | | | | | |
| --- | --- | --- | --- | --- | --- |
| **Category for introns** | **Location of introns** | ***Shiraia bambusicola*** | | ***Phaeosphaeria nodorum*** | ***Bipolaris maydis*** |
| Group I | *cox1* | IB (complete; LAGLIDADG  endonuclease) | | | IB (complete), IB (complete), I (derived, B2), IB (complete), IB  (extra insertion), IB (3', partial), IB (complete), IB (complete) |
|  | *cox2* |  |  | | IC1, I (no intron type identified), IB (complete), I (no intron type  identified) |
|  | *cox3* |  | I (derived, B1) (LAGLIDADG endonuclease) | |  |
|  | *nad1* |  | IB (complete, GIY-YIG endonuclease ) | |  |
|  | *nad2* |  |  | | IC2 |
|  | *nad3* |  |  | |  |
|  | *nad4* |  |  | |  |
|  | *nad4L* |  |  | |  |
|  | *nad5* |  | IC2 (LAGLIDADG endonuclease) | | I (no intron type identified), IC2, IB (complete), IB (complete) |
|  | *nad6* |  |  | |  |
|  | *cob* |  | IB (complete, LAGLIDADG endonuclease) | | IB (complete), ID, ID, ID, IB (complete), I (no intron type  identified), I (no intron type identified), IA, IB (complete), IB (3',  partial), I (no intron type identified), ID |
|  | *atp6* |  | IC2 (GIY-YIG endonuclease ) | |  |
|  | intergenic region |  | IC1 | |  |
|  | *rnl* |  |  | |  |
| Group II | *rns* |  |  | |  |

| **Category for introns** | **Location of introns** | ***Pyrenophora tritici-repentis*** |
| --- | --- | --- |
| Group I | *cox1* | IB (complete), IB (complete), IB (complete), IC2, IB (complete), I(derived), I (no intron type identified), IB (complete), IB (complete), IB (complete), IB (complete), I(derived), IB (complete), IB (complete), IA(5', partial), |
|  | *cox2* | ID, IC1, |
|  | *cox3* | IB (complete), IC2, I (no intron type identified), |
|  | *nad1* |  |
|  | *nad2* | IC2 (Potentially missing exon (~20)), IC2 (Potentially missing exon (~53)), I (no intron type identified) |
|  | *nad3* |  |
|  | *nad4* |  |
|  | *nad4L* |  |
|  | *nad5* | I (no intron type identified), IC2, IB (complete), ID, IB(3', partial), IB (complete), IB (complete), IB (complete), IC2, IC2, |
|  | *nad6* |  |
|  | *cob* | IB (complete), ID, ID, IB (complete), |
|  | *atp6* | IB (complete), IC2 |
|  | intergenic region |  |
|  | *rnl* |  |
| Group II | *rns* |  |

| **Category for introns** | **Location of introns** | ***Leptosphaeria maculans*** | ***Neofusicoccum parvum*** | |
| --- | --- | --- | --- | --- |
| Group I | *cox1* | I (derived, A), I (derived, B2), IB (complete), IB (complete), I (no intron type identified), IB (complete), IB (complete) |  | |
|  | *cox2* | IC1, I (no intron type identified), IC2, ID, IB (3', partial), I (no intron type identified), | ID, IB (complete) | |
|  | *cox3* | IC2, IA (5', partial), IA, ID, IB (complete), intron I (derived, B1) | |  |
|  | *nad1* | IB (3', partial), I (no intron type identified), IB (complete) | | |
|  | *nad2* | I (no intron type identified), I (no intron type identified) | IC2 | |
|  | *nad3* |  |  | |
|  | *nad4* | IC2 |  | |
|  | *nad4L* |  |  | |
|  | *nad5* | I (no intron type identified), IC2, ID, IB (complete), I (no intron type identified), IB (3', partial), IC2 | IB (complete), IC2 | |
|  | *nad6* |  |  | |
|  | *cob* | IB (complete), I (no intron type identified), IB (complete), IB (3', partial), I (no intron type identified), ID |  | |
|  | *atp6* | I (no intron type identified), IC2, IB (complete) | IB (complete) | |
|  | intergenic region |  |  | |
|  | *rnl* |  | intron I (derived, A) | |
| Group II | *rns* | intron II (domainV) | intron II (domainV) | |
