# Supplementary material for: Characterization and Phylogenetic Analysis of the Mitochondrial Genome of Shiraia bambusicola Reveals Special Features in the Order of Pleosporales
Source: PLoS One. 2015 Mar 19;10(3):e0116466. doi: 10.1371/journal.pone.0116466 (PMC4366305; doi:10.1371/journal.pone.0116466)
Supplement: S3 Table — (DOC) [file pone.0116466.s003.doc]

| **Table S3. ORFs found in mitochondrial genes from Dothideomycetes species*.** | | | | | |
| --- | --- | --- | --- | --- | --- |
| **Location of introns** | ***Shiraia bambusicola*** | ***Bipolaris maydis*** | ***Pyrenophora tritici-repentis*** | ***Leptosphaeria maculans*** | ***Neofusicoccum parvum*** |
| *cox1* | *orf352* | *orf318* | *orf324-1* | *orf324-2* | *orf321* |
| intergenic region | *orf250*, *orf262*, *orf322* and *orf564* |  | *orf493*, *orf205* and *orf243* | *orf221*, *orf207*, *orf535* and *orf158* |  |
| *Due to space constraints, ORFs in the table were only which had been characterized in the main text. | | | | | |
