# Supplementary material for: Characterization and Phylogenetic Analysis of the Mitochondrial Genome of Shiraia bambusicola Reveals Special Features in the Order of Pleosporales
Source: PLoS One. 2015 Mar 19;10(3):e0116466. doi: 10.1371/journal.pone.0116466 (PMC4366305; doi:10.1371/journal.pone.0116466)
Supplement: S4 Table — (DOC) [file pone.0116466.s004.doc]

**Table S4. Distribution of large repeat loci in the mitochondrial genome of *Shiraia bambusicola.***

| **No.** | **Type** | **Size (bp)** | **Start (Unit 1)** | **Location (Unit 1)** | **Start (Unit 2)** | **Locatin (Unit 2)** |
| --- | --- | --- | --- | --- | --- | --- |
| P1 | P | 485 | 14240 | CDS (atp6) | 24719 | CDS (atp6) |
| P2 | P | 333 | 13454 | IGS (trnL, trnQ) | 26431 | IGS (trn M, trnH) |
| P3 | P | 218 | 27714 | IGS (orf250, nad3) | 38805 | IGS (trnR, cox1) |
| P4 | P | 110 | 13794 | IGS (trnM, atp6) | 26167 | IGS (orf262, trnM) |
| P5 | P | 103 | 4144 | IGS (cox2, trnN) | 35209 | IGS (trnV, trnN) |
| P6 | P | 99 | 14203 | CDS (atp6) | 25102 | CDS (atp6) |
| P7 | P | 81 | 5193 | IGS (nad6, trnV) | 35078 | IGS (trnK, trnV) |
| P8 | P | 72 | 5200 | CDS (trnV) | 24491 | CDS (trnV) |
| F1 | F | 77 | 24491 | CDS (trnV) | 35080 | CDS (trnV) |
| P9 | P | 72 | 27996 | IGS (orf250, nad3) | 38645 | IGS (trnR, cox1) |
| F2 | F | 67 | 17856 | CDS (nad4) | 17880 | CDS (nad4) |
| F3 | F | 63 | 24501 | CDS (trnV) | 35090 | CDS (trnV) |
| F4 | F | 50 | 18071 | CDS (nad4) | 18119 | CDS (nad4) |
| P10 | P | 53 | 28015 | IGS (orf250, nad3) | 38645 | IGS (trnR, cox1) |
| P11 | P | 44 | 0 | IGS (trnR, cox1) | 27710 | IGS (orf250, nad3) |
| F5 | F | 43 | 24521 | CDS (trnV) | 35110 | CDS (trnV) |
| P12 | P | 52 | 27896 | IGS (orf250, nad3) | 38789 | IGS (trnR, cox1) |
| F6 | F | 40 | 0 | IGS (trnR, cox1) | 38983 | IGS (trnR, cox2) |
| R1 | R | 38 | 31005 | IGS (nad2, cox3) | 31005 | IGS (nad2, cox3) |
| F7 | F | 43 | 17856 | CDS (nad4) | 17904 | CDS (nad4) |
| P13 | P | 40 | 3914 | IGS (cox2, trnN) | 3914 | IGS (cox2, trnN) |
| P14 | P | 40 | 16513 | IGS (nad1, orf322) | 16513 | IGS (nad1, orf322) |
| R2 | R | 33 | 16452 | IGS (nad1, orf323) | 16452 | IGS (nad1, orf323) |
| P15 | P | 39 | 14686 | CDS (atp6) | 24679 | CDS (atp6) |
| F8 | F | 39 | 30995 | IGS (nad2, cox3) | 31006 | IGS (nad2, cox3) |
| P16 | P | 36 | 14172 | CDS (atp6) | 25193 | CDS (atp6) |
| P17 | P | 32 | 13896 | IGS (trnM, atp6) | 26144 | IGS (orf262, trnM) |
| F9 | F | 41 | 1094 | CDS (cox1) | 1136 | CDS (cox1) |
| F10 | F | 35 | 20070 | CDS (trnK) | 35126 | CDS (trnK) |
| F11 | F | 35 | 21877 | IGS (cob, nad5) | 21901 | IGS (cob, nad5) |
| P18 | P | 35 | 28033 | IGS (org250, nad3) | 38645 | IGS (trnR, cox1) |
| R3 | R | 31 | 31014 | IGS (nad2, cox3) | 31014 | IGS (nad2, cox3) |
| R4 | R | 37 | 30995 | IGS (nad2, cox3) | 31005 | IGS (nad2, cox3) |
| F12 | F | 39 | 1076 | CDS (cox1) | 1118 | CDS (cox1) |
| P19 | P | 39 | 14315 | CDS (atp6) | 25233 | CDS (atp6, orf262) |
| F13 | F | 39 | 25050 | CDS (atp6) | 25233 | CDS (atp6, orf262) |
| F14 | F | 30 | 20002 | CDS (trnK) | 24491 | CDS (trnV) |
| P20 | P | 33 | 13754 | IGS (trnM, atp6) | 26282 | IGS (orf262, trnM) |
| F15 | F | 34 | 1971 | CDS (cox1) | 2009 | CDS (cox1) |
| F16 | F | 34 | 3887 | IGS (cox2, trnN) | 3911 | IGS (cox2, trnN) |
| R5 | R | 36 | 21877 | IGS (cob, nad5) | 21885 | IGS (cob, nad5) |
| R6 | R | 36 | 21883 | IGS (cob, nad5) | 21900 | IGS (cob, nad5) |
| P21 | P | 33 | 5193 | IGS (nad6, trnV) | 20070 | CDS (trnK) |
| F17 | F | 30 | 3972 | IGS (cox2, trnN) | 3982 | IGS (cox2, trnN) |
| P22 | P | 30 | 5242 | CDS (trnV) | 20002 | CDS (trnK) |
| T1 | T | 42*2.5 | 1077 | CDS (orf352) |  |  |
| T2 | T | 38*2.6 | 1944 | CDS (orf352) |  |  |
| T3 | T | 29*2.3 | 3795 | IGS (cox2, trnN) |  |  |
| T4 | T | 24*2.2 | 3888 | IGS (cox3, trnN) |  |  |
| T5 | T | 20*2.3 | 4394 | IGS (trnN, nad6) |  |  |
| T6 | T | 60*1.9 | 6264 | IGS (trnD, trnS) |  |  |
| T7 | T | 21*2.3 | 6589 | IGS (trnS, trnW) |  |  |
| T8 | T | 18*2.8 | 6698 | IGS (trnS, trnW) |  |  |
| T9 | T | 18*2.9 | 7430 | IGS (trnS, trnP) |  |  |
| T10 | T | 19*4.9 | 7456 | IGS (trnS, trnP) |  |  |
| T11 | T | 32*2.0 | 7747 | IGS (trnP, rnl) |  |  |
| T12 | T | 17*2.5 | 13155 | IGS (trnF, trnL) |  |  |
| T13 | T | 27*3.2 | 13273 | CDS (trnL) |  |  |
| T14 | T | 48*2.7 | 17857 | CDS (nad4) |  |  |
| T15 | T | 48*2.2 | 18072 | CDS (nad4) |  |  |
| T16 | T | 22*2.0 | 19512 | CDS (nad5) |  |  |
| T17 | T | 57*2.1 | 20134 | IGS (trnK, cob) |  |  |
| T18 | T | 38*2.4 | 21781 | IGS (cob, nad5) |  |  |
| T19 | T | 24*2.8 | 21878 | IGS (cob, nad5) |  |  |
| T20 | T | 66*2.2 | 27518 | CDS (orf250) |  |  |
| T21 | T | 21*3.4 | 30970 | IGS (nad2, cox3) |  |  |
| T22 | T | 15*4.1 | 34734 | CDS (rps3) |  |  |
| T23 | T | 15*3.4 | 35144 | CDS (trnV) |  |  |
| T24 | T | 21*2.4 | 35666 | IGS (trnY, trnL) |  |  |
| T25 | T | 21*2.4 | 35829 | IGS (trnY, trnL) |  |  |
